# Targeting menin: a promising therapeutic strategy for susceptible acute leukemia subtypes

**DOI:** 10.1038/s41392-023-01627-w

**Published:** 2023-10-11

**Authors:** Pietro Di Fazio

**Affiliations:** Department of Visceral, Thoracic and Vascular Surgery, Philipps University of Marburg, Baldingerstrasse, 35043 Marburg, Germany

**Keywords:** Haematological cancer, Haematological cancer, Molecular medicine, Drug development

Two recent studies published in *Nature* focused on the clinical efficacy of the menin inhibitor revumenib (SNDX-5613) in KMT2A (histone-lysine *N*-methyltransferase 2A)-rearranged or NPM1 (Nucleophosmin1)-mutant acute myeloid leukemia.^1,2^ These studies indicate that menin inhibition and its acquired resistance to long-term exposure to revuminib are key points for future treatment, emphasizing the role of menin as a challenging and promising target for the current treatment of leukemia.

KMT2A mutations arise in 80% of acute lymphoblastic leukemia (ALL) and, together with mutations related to NPM1, in 30% of acute myeloid, lymphoid or mixed phenotype leukemia (AML). KMT2A is a fine epigenetic regulator that binds the promoter sequences of the HOX (Homeobox) genes deputed for the proliferation of the lymphoblastic cells. These genes are normally not expressed in differentiated cells. Mutations occurring KMT2A cause the stabilization of the binding with menin, which is co-binding the promoter regions of the HOX. Thus triggering the transcription of the HOX genes and their DNA-binding-cofactor MEIS1 (Homeobox Protein Meis1), the uncontrolled proliferation and the further leukemic transformation. Besides, in AML with mutated NPM1, the KMT2A-menin synergy prompts HOX and MEIS1-mediated transcription of leukemia associated genes.^3^

**Fig. 1:**
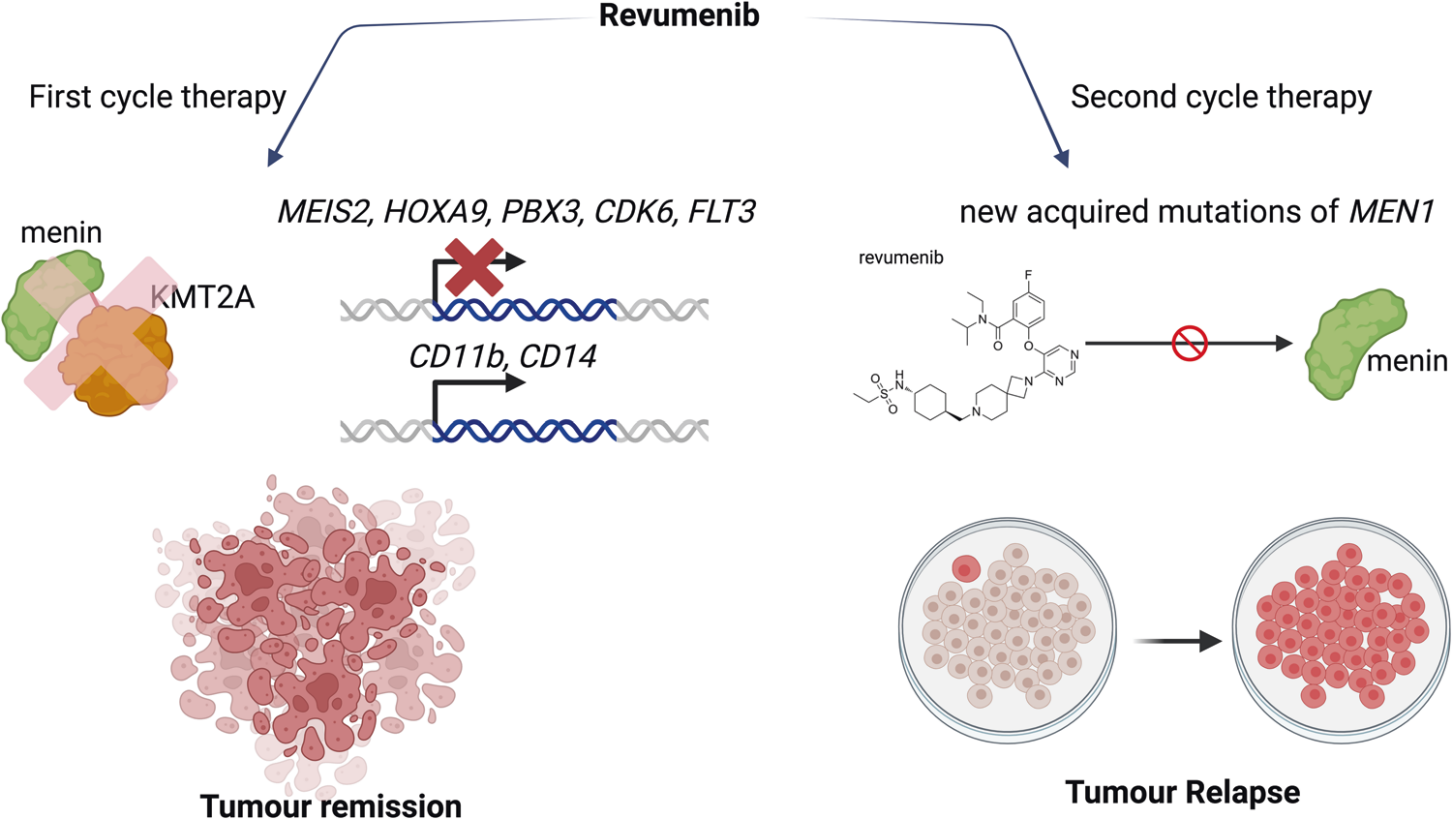
Efficacy of revumenib in clinical trial and its loss of affinity for mutated menin. The first cycle of therapy with revumenib inhibits the binding between menin and KMT2A, thus leading to the suppression of leukemia drivers, over-expression of differentiation related genes, block of tumor progression, and further remission. The second cycle therapy causes the acquisition of mutation within the MEN1 gene that lead to a loss of the affinity of revumenib for menin and tumor relapse. The chemical structure of revumenib is illustrated with ChemDraw 20.0. The figure has been created with BioRender.com

The study group of Issa and colleagues performed the first clinical trial with the menin inhibitor revumenib in subjects affected by leukemia harboring a rearranged KMT2A or mutated NPM1. The blockade of the menin-KMT2A synergy is able to disrupt the binding of the oncogenic KMT2A wild type or the fusion complexes on the chromatin. This study was able to identify the safety, the maximum tolerated dose, the recommended phase dose 2, pharmacokinetic and pharmacodynamics (Fig. 1).

This study evidenced that the clinical administration of revumenib was able to downregulate the expression of MEIS2, HOXA9, pre-B cell leukemia transcription factor 3 (PBX3), the cyclin-dependent kinase 6 (CDK6), and fms-like tyrosine kinase 3 (FLT3). Instead, the genes related to differentiation-like integrin alpha M (CD11b) and CD14 were overexpressed.

Despite the progress in the treatment of childhood acute leukemia, infant KMT2Ar acute leukemias have remained a therapeutic challenge with high rates of resistance to therapy with multi-agent chemotherapy. This first-in human clinical trial provided clinical indication of the possibility to inhibit menin with an oral selective therapy. Furthermore, this was the first evidence of an epigenetic treatment being able to dissociate the protein complexes from the chromatin thus leading to remissions in patients affected by acute leukemia. The deep molecular remission was associated by minimal toxicities in children and adults participants.^1^

However, acquired mutations of the encoding sequence of the MEN1 gene cause an altered menin, thus preventing the binding of revumenib and can lead to clinical relapse.

This aspect has been further investigated by the group of Perner and colleagues.^2^ In particular, the study focused on the patients enrolled in the previously mentioned clinical trial (AUGMENT-101) that showed, after an initial response to revumenib, a relapse of acute leukemia. Next generation selective sequencing of bone marrow samples from these subjects evidenced a largely stable prospect of well-known leukemia drivers. Interestingly, the somatic mutations within MEN1 gene were detected at time of relapse on revumenib. The study further focused on detecting such MEN1 somatic mutations in specimens available from other patients included in the trial. The analysis, performed by droplet digital PCR, highlighted that several other patients developed somatic mutations of the MEN1 gene that were not detectable before. In particular, such mutations harbored MEN1 during the administration of revumenib after approximately two cycles of therapy. Similar results were observed in experiments conducted with patient-derived xenografts. The research group identified several mutations of the MEN1 gene in those xenografts that developed after a prolonged drug exposure. Thus suggesting that the administration of revumenib induced de novo mutations. The identified recurrent somatic mutations, which affect the M327, G331, T349, and S160, are different from known variants that counteract the tumor suppressive activity of menin in MEN1 syndrome and they have been described in this study for the first time.

This study reported, for the first time, that the mutations occurring at MEN1 gene caused a change in the binding pocket amino acids sequence of the menin protein that impedes the binding with revumenib. Thus perturbing the inhibitory activity of revumenib and leading to a relapse of the tumor.^2^

The remark that such alterations confer resistance across different menin inhibitors suggests that next-generation compounds are strongly needed to overcome the cellular ability to activate mechanism of resistance. Structure guide drug design of substances that bind to KMT2Ar circumventing its binding with the mutated form of menin could represent an effective strategy for the advanced therapy of patients affected by ALL and AML.

The ability of menin to regulate the transcriptional machinery does not only rely on the interaction with KMT2A and the indirect effect of NPM1. Besides its oncogenic activity in KMT2Ar and mutated NPM1 leukemia, menin can directly interact with chromatin histones too. In particular, menin is able to read the nucleosomal methylated H3K79me2.^4^ This recently discovered new activity of menin has not been investigated in tumor yet.

Its function could influence both its tumor suppressor and oncogenic activity and could be a potential target for the next-generation therapy. Up to now, the mutations occurring at MEN1 gene, which were identified in the study of Perner and colleagues, could exert a role also in the affinity binding with the H3K79me2.

Nonetheless, recent discoveries highlighted the neddylation as a mechanism being able to modulate menin activity. Specifically, this ubiquitination dependent mechanism is regulated by the involvement of the protein NEDD8 and triggers, in pancreatic neuroendocrine neoplasia, menin degradation because of an over-expression of neddylation-related enzymes.^5^ It could not be excluded that such degradation mechanism can directly affect menin role in several pathways and not only at transcriptional level but also in the cytosolic compartment, thus affecting cell death related processes.

The discovery of new processes and epigenetic modulation connecting with the protein encoded by the MEN1 gene should be included in the next-generation designing of specific drugs direct against players of neddylation and further improve the design of substances being able to act at the epigenetic level of MEN1/menin modulation.
